# A113 COVID-19 VACCINATIONS IN IBD PATIENTS: PATIENT KNOWLEDGE AND PERCEPTIONS

**DOI:** 10.1093/jcag/gwad061.113

**Published:** 2024-02-14

**Authors:** A Saunders, L Hill, D Armstrong, J Marshall, N Narula, N Pai, U Chauhan

**Affiliations:** McMaster University Michael G DeGroote School of Medicine, Hamilton, ON, Canada; McGill University Health Centre, Montreal, QC, Canada; Medicine, McMaster University Faculty of Health Sciences, Hamilton, ON, Canada; Medicine, McMaster University Faculty of Health Sciences, Hamilton, ON, Canada; Medicine, McMaster University Faculty of Health Sciences, Hamilton, ON, Canada; Medicine, McMaster University Faculty of Health Sciences, Hamilton, ON, Canada; Hamilton Health Sciences, Hamilton, ON, Canada

## Abstract

**Background:**

The IBD population has historically suffered from a below average uptake of vaccinations which raises concern for COVID-19 vaccine acceptance in this population. Patients report IBD-specific reasons for COVID-19 vaccine hesitancy including fear of vaccine-related IBD flare up, a desire for IBD-specific data regarding COVID-19 vaccine safety and efficacy, and worry about current drugs affecting vaccine efficacy. Additionally, IBD patients tend to report greater fear of COVID-19, concern about the impact of their medications on COVID-19 disease, and overall need for COVID-19 information.

**Aims:**

To explore patients’ education, knowledge, and perceptions of COVID-19 vaccination with the aim of lowering COVID-19 vaccine hesitancy, improving knowledge of COVID-19 vaccines, and identifying outstanding barriers to COVID-19 vaccination uptake.

**Methods:**

Study participants are patients diagnosed with IBD recruited from the Adult IBD Clinic at McMaster University Medical Centre between June 2022 and May 2023. Quantitative questionnaires were distributed to participants following receipt of informed consent at routine clinics and were offered in paper format. To describe the study population, the following descriptive statistics were performed: means and standard deviation for continuous variables, distributions (n, %) for dichotomous and categorical variables.

**Results:**

In total, 236 participants were surveyed. The sample is predominantly female (61%) and most participants are diagnosed with Crohn’s disease (63.9%). The vast majority of patients have received at minimum 1 dose of a COVID-19 vaccine (92.1%). Reported reasons for vaccination included COVID-19 vaccine being available free of cost (52.3%), recommendation from a healthcare professional (51.9%), and feeling as though the benefit of the vaccine outweighs the risks (51.6%). Most patients reported having heard of negative vaccine experiences online (75.8%) including mild (27.6%) and severe (30.8%) adverse reactions. Despite this, participants viewed vaccines as effective (83%), protective towards the individual (75.5%), and safe (68.1%). Healthcare providers were regarded as a very significant influence in terms of COVID-19 vaccine information (65.6%) and were overwhelmingly viewed as a more reliable source than mass media (91.3%). Participants did not perceive an increased risk of COVID-19 infection, COVID-19 related serious illness, or COVID-19 vaccine related adverse effects due to their IBD or IBD treatments. Participants also did not show reluctance to vaccinate due to their IBD or IBD treatments.

**Conclusions:**

The present study provides insight into the perceptions and knowledge of Canadian IBD patients as it relates to COVID-19 vaccines. Importantly, the results highlight the crucial role of the healthcare provider on vaccination uptake and acceptance.

**Funding Agencies:**

None

